# One-year real-world outcomes after vericiguat initiation in heart failure: The ROVER-Japan cohort study

**DOI:** 10.1016/j.ijcha.2026.101900

**Published:** 2026-03-13

**Authors:** Makiko Takeichi, Shun Kohsaka, Kotaro Nochioka, Christoph Ohlmeier, Alexander Michel, Katsiaryna Holl, Hiroki Yamamoto, Yoshifumi Arita, Seok-Won Kim, Suguru Okami

**Affiliations:** a Medical Affairs & Pharmacovigilance, Bayer Yakuhin, Ltd., 2-4-9 Umeda, Kita-ku, Osaka 530-0001, Japan; b Department of Cardiology, Keio University School of Medicine, 35 Shinanomachi, Shinjuku-ku, Tokyo 160-8582, Japan; c Department of Cardiovascular Medicine, Tohoku University Hospital, 1-1 Seiryo-machi, Aoba-ku, Sendai, Miyagi 980-8574, Japan; d Real-World Evidence Center of Excellence, Bayer AG, NNOC, Building S102, 13353 Berlin, Germany; e Real-World Evidence Center of Excellence, Bayer Consumer Care AG, Peter Merian Straße 84, CH-4052 Basel, Switzerland; f Global Medical and Evidence, Bayer AG, 13342 Berlin, Germany; g Real-World Evidence Solutions & HEOR, IQVIA Solutions Japan G.K., 4-10-18 Takanawa, Minato-ku, Tokyo 108-0074, Japan

**Keywords:** Vericiguat, Heart failure, Database, Japan

## Abstract

**Background:**

Vericiguat, a soluble guanylate cyclase stimulator, is recommended for patients with chronic heart failure (HF) who recently worsened despite guideline-directed medical therapy. However, there is limited data on its real-world utilization and outcomes. The ROVER-Japan study examined prescribing patterns and prognosis among Japanese patients who initiated vericiguat in routine practice.

**Methods and results:**

This retrospective cohort study analyzed secondary data from a Japanese hospital administrative database. Among adult patients diagnosed with HF, 4936 patients who initiated a starting dose of vericiguat (2.5 mg/day) on top of any fundamental HF therapy between September 15, 2021, and July 31, 2024, were included. Patients were followed for up to one year or until death, loss to follow-up, or the end of the study period. The mean age of the included patients was 75.4 (standard deviation 12.4) years. At vericiguat initiation, 60.4% of the patients had a recent worsening HF event, defined as HF hospitalization (HFH) in the previous six months or outpatient intravenous diuretics in the previous three months. Quadruple guideline-directed therapy was administered to 29.6% of patients at baseline. One year after treatment initiation, 95.7% of patients had a medication possession ratio ≥ 80%, and 66.6% continued vericiguat treatment. Natriuretic peptide levels and diuretic use decreased, while eGFR remained stable after vericiguat initiation. The one-year cumulative incidence of composite of cardiovascular death or HFH was 29.8%.

**Conclusions:**

This study provides real-world insights into the contemporary use and outcomes of vericiguat in a large, diverse HF population.

## Highlights

• Vericiguat was administered to a broader population compared to a clinical trial.
• Vericiguat was often initiated in patients not on quadruple foundational therapy.
• Multiple heart failure medications were adjusted around vericiguat initiation.
• Challenges remain for persistence of heart failure therapy in real-world settings.
• One-year cumulative incidence of composite outcome was similar to clinical trial.

## Graphical abstract

**Figure:**
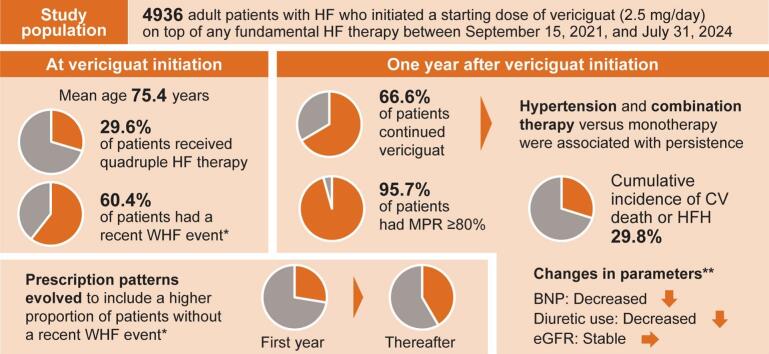
One-year follow-up of patients with HF after initiating vericiguat. *Defined as HFH in the previous six months or outpatient intravenous diuretics in the previous three months. **Parameters were assessed at pre- (day -90 to day -1) and post-vericiguat initiation periods (day 1 to day 90, day 91 to day 180). BNP, B-type natriuretic peptide; CV, cardiovascular; eGFR, estimated glomerular filtration rate; HF, heart failure; HFH, heart failure hospitalization; MPR, medication possession ratio; WHF, worsening heart failure.

## 1 Introduction

Heart failure (HF) is a complex multifactorial clinical syndrome defined by structural or functional ventricular impairment that compromises filling or ejection, producing dyspnea, fatigue, cough, fluid retention, elevated jugular venous pressure, pulmonary crackles, and peripheral edema [1]. Recurrent episodes of worsening HF (WHF) accelerate disease progression and carry a high short-term mortality risk [2]. For HF with reduced ejection fraction (HFrEF), contemporary guidelines endorse four “foundational” drug classes (i.e., renin-angiotensin system [RAS] inhibitors, beta-blockers, mineralocorticoid receptor antagonists [MRAs], and sodium-glucose cotransporter 2 inhibitors [SGLT2is]) as the cornerstone of therapy [3], [4], [5].

Vericiguat, a soluble guanylate cyclase (sGC) stimulator, targets and restores the impaired nitric oxide-sGC-cyclic guanosine monophosphate (NO-sGC-cGMP) pathway in HF [6], [7], addressing a mechanism of action not covered by the four foundational therapies. Based on the results of the phase III VICTORIA trial [8], vericiguat was approved by healthcare authorities in the United States, Japan, and the European Union in 2021 and received Class IIa or IIb guideline recommendation for patients with HFrEF who have experienced a recent WHF event [3], [4], [5].

However, real-world evidence on vericiguat remains limited, confined to studies with modest patient numbers [9], [10], [11], [12], [13], [14], [15], [16]. There remains a need for a deeper understanding of its usage patterns and patient prognosis in routine practice. The Real-world Outcomes of patients treated with VEriciguat in Japanese Routine care (ROVER-Japan) study leverages one of the country’s largest hospital administrative databases to characterize the one-year prescribing patterns and clinical outcomes after initiating vericiguat in routine practice.

## 2 Methods

### 2.1 Study design

The ROVER-Japan study (NCT06697353) was a retrospective, observational cohort study using secondary data from a Japanese hospital administrative database provided by Medical Data Vision Co., Ltd. (MDV; Tokyo, Japan). The data period spanned from September 1, 2016, to July 31, 2024, which includes the baseline and eligibility assessment periods. The patient enrollment period was from September 15, 2021 (i.e., the date of commercial launch in Japan) to July 31, 2024. The primary endpoint was to document the incidence of composite events of cardiovascular (CV) death or HF hospitalization (HFH). This was a descriptive study intended to characterize real-world outcomes, rather than to test a specific hypothesis. The study was conducted in accordance with the Declaration of Helsinki. Ethical approval for this study was obtained from the independent ethics committee of the specified nonprofit organization, MINS, on October 17, 2024 (Approval No. 240216). In Japan, informed consent does not apply to the use of de-identified secondary data in accordance with the Ethical Guidelines for Medical and Biological Research Involving Human Subjects. The use of de-identified data adhered to local regulations, including the Personal Information Protection Law.

### 2.2 Study population

This study included patients aged ≥ 18 years who initiated a starting dose of vericiguat (2.5 mg/day) in addition to any dose of HF foundational therapy between September 15, 2021 and July 31, 2024, with a confirmed diagnosis of HF between March 1, 2021 and July 31, 2024, before the index date. Patients both with and without a recent WHF event at baseline were included, since the approved indication for vericiguat in Japan is for chronic heart failure in patients receiving standard therapy and is not restricted to those with a recent WHF event. Patients were required to have at least 180 days of observability before the index date and at least one day of follow-up. The index date was the date of vericiguat administration initiation (day 0). The inclusion and exclusion criteria are summarized in Supplementary Table 1. Baseline characteristics were assessed using medical records from 180 days before the index date. Some variables (e.g., diagnosis, comorbidities, and procedures) were collected from all study periods before the index date. Baseline HF therapy was assessed within 90 days before the index date. Patients were followed up for a maximum of one year after the index date; follow-up ended upon patient death, loss to follow-up, or the end of the study period. Patients were categorized into prespecified subgroups based on the presence or absence of a recent WHF event, index date, up-titration level of vericiguat at day 90, adherence to vericiguat within 90 days, persistence to vericiguat within 90 days, presence of chronic kidney disease, age, and body mass index (BMI). In Japan, new pharmaceuticals are generally subject to a 14-day prescription limit during the first year, meaning that a maximum of 14 days of medication can be prescribed at one time. Consequently, we defined subgroups based on index date as follows: the early group, which initiated vericiguat treatment within the first year after the commercial launch (from September 2021 to August 2022), and the late group, which initiated vericiguat treatment one year later (from September 2022 to July 2024). The definitions of the variables and dosage categories are summarized in Supplementary Tables 2 and 3, respectively.

### 2.3 Treatment patterns of HF foundational therapies

The foundational therapies for HF included RAS inhibitors (angiotensin-converting enzyme inhibitors [ACEis], angiotensin receptor blockers [ARBs], or angiotensin receptor-neprilysin inhibitor [ARNI]), beta-blockers, MRAs, and SGLT2is. The day-to-day patterns of dosing from day −90 to day 365, the cumulative incidence of prescription from day −90 to day 0 and from day 0 to day 365, and the number of HF foundational therapies at predefined periods (i.e., day −90 to day −1, day 0 to day 90, day 91 to day 180, day 181 to day 270, day 271 to day 365) were assessed in patients with one year of follow-up (n = 1798).

### 2.4 Patterns of vericiguat dose titration, adherence, and persistence

The day-to-day patterns of vericiguat dose titration and treatment adherence were assessed from the index date until day 365. Measures of adherence included medication possession ratio (MPR) and proportion of days covered (PDC). Persistence was defined as the number of patients who received continuous treatment on day 365. Continuous treatment was assumed if a subsequent prescription occurred within days of the previous prescription or during a grace period of 90 days thereafter. These analyses were conducted in patients with one year of follow-up. The factors associated with one-year persistence of vericiguat were explored using multivariable logistic regression models in patients with one year of follow-up and without missing value in the variables of interest (n = 1092).

### 2.5 Changes in laboratory and clinical parameters

N-terminal pro-B-type natriuretic peptide (NT-proBNP), BNP, estimated glomerular filtration rate (eGFR), hemoglobin, number of HFHs per patient, percentage of patients with diuretic use, and daily dose of loop diuretics (presented as furosemide equivalents: 40 mg of furosemide was considered equivalent to 60 mg of azosemide or 20 mg of torsemide) were assessed at pre- (i.e., day −90 to day −1) and post-vericiguat initiation periods (i.e., post-1, day 1 to day 90; post-2, day 91 to day 180). The eGFR was calculated for patients aged ≥ 18 years using the equation for Japanese patients (i.e., eGFR = 194 × [serum creatinine]^-1.094^ × [age]^-0.287^ × 0.739 [if female]) [17]. This equation is stated in Japanese guidelines for chronic kidney disease and is used in local clinical practice [18]. For cases where the calculation was not possible due to missing variables (e.g., serum creatinine), the eGFR value recorded by each institution was used. As this study utilized de-identified secondary data, the method used to determine these recorded values could not be retrospectively verified for individual patients. If a patient had multiple records of laboratory parameters, the results reported closest to the index date (pre) and the results last reported during the time window (post-1 and post-2) were used. Only patients with at least one test result in each period (pre-, post-1, and post-2) were included in the analysis of each laboratory test. The number of HFHs per patient, percentage of patients using diuretics, and daily dose of loop diuretics were analyzed in patients with at least 180 days of follow-up. The same analyses were conducted among patients whose HF therapeutics (i.e., ACEi, ARB, ARNI, beta-blocker, MRA, and SGLT2i) remained unchanged, defined as those whose combination of HF medication did not vary across any of the three specified periods (pre-, post-1, and post-2).

### 2.6 Clinical outcomes

Composite of CV death or HFH, CV death, HFH, composite of all-cause death or HFH, and all-cause death were assessed in all included patients. The earliest event of each outcome was used in the calculations. The definitions of the clinical outcomes are summarized in Supplementary Table 4.

### 2.7 Statistical analysis

Continuous variables are reported as means and standard deviation (SD) or medians and interquartile range. Categorical variables are summarized as numbers and proportions. The numbers and proportions of patients with missing data were reported. No imputations were performed for this observational study. The cumulative percentages of patients initiating HF foundational therapies and those who experienced clinical outcomes were calculated using the Kaplan–Meier method. Landmark analysis after day 90 was performed to calculate cumulative incidences of clinical outcomes in subgroups based on up-titration, adherence and persistence. To explore factors associated with persistence, odds ratios (OR) were calculated using multivariable logistic regression models with p-values from the Wald test. Model covariates were selected from patient characteristic variables, with those highly correlated with each other removed based on a correlation matrix to account for potential multicollinearity. Changes in parameters over time were statistically tested using repeated-measures analysis of variance (ANOVA) or the Friedman test. For the Bonferroni-adjusted post hoc pairwise comparisons of these parameter changes, the *t*-test was used for continuous variables and the McNemar test was used for binary variables. The adjusted p-values were calculated by multiplying the original p-value by 3. If the adjusted p-value exceeded 1, it was capped at 1. To investigate the impact of a recent WHF event on clinical outcomes, hazard ratios (HRs) were calculated using the Cox proportional hazards model adjusted for baseline covariates, and the p-values were calculated using the Wald test. For all computations, data analyses and generation of tables, listings and data for figures were performed using Statistical Analysis System® (SAS) version 9.4 or higher (SAS Institute, Cary, NC, USA) or using R version 3.6 or higher.

## 3 Results

### 3.1 Patient characteristics

A total of 4936 patients with HF who were newly prescribed vericiguat between September 15, 2021, and July 31, 2024, were included in this study. The patient characteristics are summarized in Table 1. The mean patient age was 75.4 (SD 12.4) years, and 32.6% were female. At vericiguat initiation, 60.4% of patients had a recent WHF event, defined as HFH in the previous six months or outpatient intravenous diuretics in the previous three months, whereas 39.6% of patients did not. Among baseline comorbidities, 86.3% of patients had hypertension, 35.1% had chronic kidney disease, 65.1% had diabetes mellitus, and 66.1% had coronary artery disease. RAS inhibitor, beta-blocker, MRA, and SGLT2i were prescribed within 90 days before the index date in 66.5%, 82.1%, 64.4%, and 63.7% of patients, respectively. Only 29.6% of patients received quadruple therapy.

**Table 1:** Patient characteristics.

**Characteristic**		**Overall** **(N = 4936)**
Age, years		75.4 (SD 12.4)
Female sex		1608 (32.6)
BMI, kg/m^2^		22.9 (SD 4.6)
	Missing	1683 (34.1)
Recent WHF event		2983 (60.4)
	HFH in the previous 3 months	2672 (54.1)
	HFH in the previous 3–6 months	191 (3.9)
	Outpatient IV diuretics in the previous 3 months	120 (2.4)
Prior WHF event		3818 (77.4)
Inpatient initiation of vericiguat		2684 (54.4)
Baseline comorbidities		
	Hypertension	4260 (86.3)
	Chronic kidney disease	1731 (35.1)
	Diabetes mellitus	3211 (65.1)
	Atrial fibrillation	2796 (56.6)
	Coronary artery disease	3264 (66.1)
	Myocardial infarction	1553 (31.5)
	Peripheral artery disease	1271 (25.7)
	Stroke	643 (13.0)
	COPD	330 (6.7)
	Anemia	1808 (36.6)
	Hyperkalemia	827 (16.8)
Dialysis		82 (1.7)
Cardiovascular procedures		
	Biventricular pacemaker	302 (6.1)
	ICD	367 (7.4)
HF foundational therapies		
	ACEi or ARB	864 (17.5)
	ARNI	2616 (53.0)
	ACEi, ARB, or ARNI	3282 (66.5)
	Beta-blocker	4054 (82.1)
	MRA	3178 (64.4)
	SGLT2i	3143 (63.7)
Number of HF foundational therapies*		
	Monotherapy	729 (14.8)
	Dual therapy	1153 (23.4)
	Triple therapy	1594 (32.3)
	Quadruple therapy	1460 (29.6)

Data are presented as n (%) or mean (SD). WHF event was defined as HFH or outpatient intravenous diuretics. *HF foundational therapies: RAS inhibitor (ACEi, ARB, or ARNI), beta-blocker, MRA, and SGLT2i. ACEi, angiotensin-converting enzyme inhibitor; ARB, angiotensin receptor blocker; ARNI, angiotensin receptor-neprilysin inhibitor; BMI, body mass index; COPD, chronic obstructive pulmonary disease; HF, heart failure; HFH, heart failure hospitalization; ICD, implantable cardioverter defibrillator; IV, intravenous; MRA, mineralocorticoid receptor antagonist; RAS, renin-angiotensin system; SD, standard deviation; SGLT2i, sodium-glucose cotransporter 2 inhibitor; WHF, worsening heart failure.

### 3.2 Treatment patterns of HF foundational therapies before and after vericiguat initiation

**Fig. 1:**
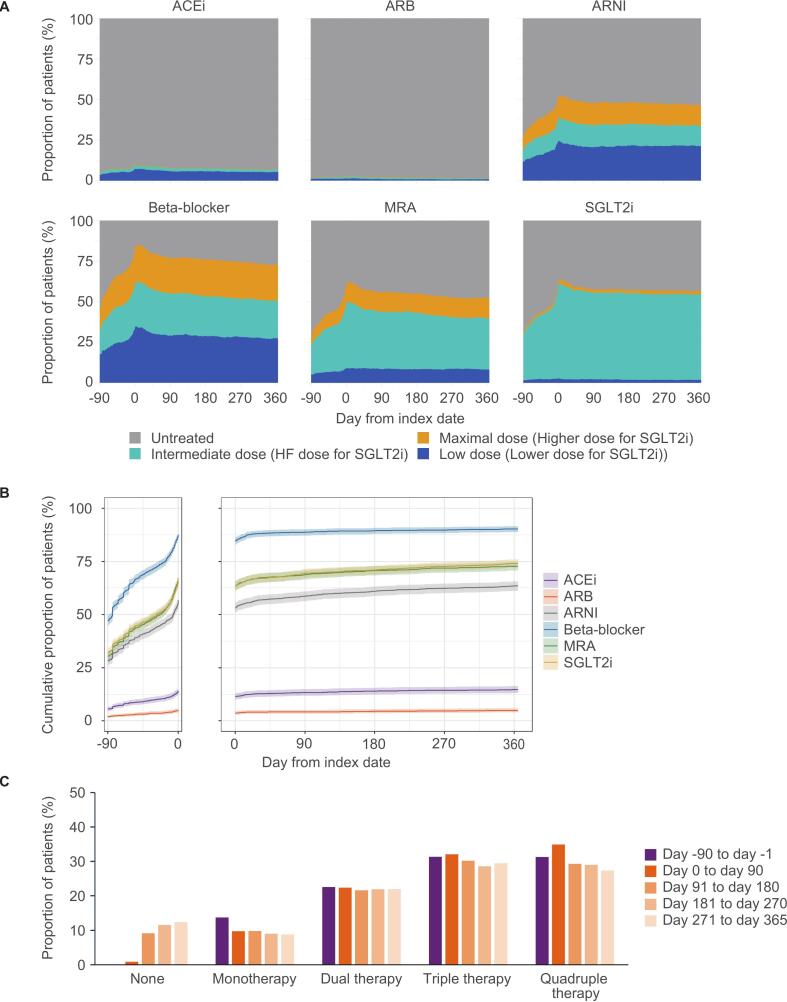
Treatment patterns of HF foundational therapies before and after vericiguat initiation. A, Dosage of HF therapeutics from day −90 to day 365. B, Cumulative proportions of patients with prescriptions of HF therapeutics from day −90 to day 0 and from day 0 to day 365. C, Number of HF foundational therapies (RAS inhibitor [ACEi, ARB or ARNI], beta-blocker, MRA and SGLT2i) administered from day −90 to day −1, day 0 to day 90, day 91 to day 180, day 181 to day 270, and day 271 to day 365. All analyses were conducted in patients with one year of follow-up (n = 1798). The shaded area in B represents the 95% confidence interval. ACEi, angiotensin-converting enzyme inhibitor; ARB, angiotensin receptor blocker; ARNI, angiotensin receptor-neprilysin inhibitor; HF, heart failure; MRA, mineralocorticoid receptor antagonist; RAS, renin-angiotensin system; SGLT2i, sodium-glucose cotransporter 2 inhibitor.

Assessment of the treatment patterns of HF therapies from day −90 to day 365 showed that the proportion of patients with prescription of ARNI, beta-blocker, MRA, and SGLT2i increased before vericiguat initiation, peaked around day 0, and gradually decreased thereafter (Fig. 1, A). The proportion of patients with prescription of ACEi and ARB were low. The cumulative prescriptions indicated that in addition to the increase in prescriptions before vericiguat initiation, prescriptions also increased slightly following vericiguat initiation (Fig. 1, B). The changes in the number of concomitant HF foundational therapies are shown in Fig. 1, C.

### 3.3 Vericiguat dose titration, adherence, and persistence

**Fig. 2:**
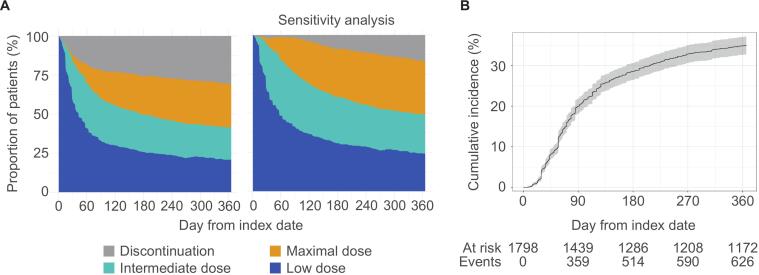
Dose titration patterns of vericiguat over one year following treatment initiation. A, Day-to-day titration patterns of vericiguat. The right panel indicates sensitivity analysis in the subgroup of patients that continued receiving vericiguat for more than three months. B, Cumulative incidence of up-titration to the maximal daily dose of vericiguat. Both A and B were analyzed in patients with one year of follow-up (n = 1798). The shaded area in B represents the 95% confidence interval.

The dose titration patterns of vericiguat over one year following treatment initiation showed vericiguat up-titration in 50.1% of patients on day 90, and 52.5% and 52.3% on day 180 and 365, respectively (Table 2 and Fig. 2, A, left panel). The percentages of patients with up-titration to the maximal daily dose on day 90, 180, and 365 were 19.0%, 26.3%, and 30.0%, respectively. The cumulative incidence plot indicated that most of the up-titration to the maximal daily dose occurred before day 180 (Fig. 2, B). Regarding adherence, 95.7% of patients had an MPR ≥ 80%, whereas 68.3% of patients had a PDC ≥ 80%. The one-year continuation rate of vericiguat was 66.6%. Among the patients who discontinued vericiguat, 66.7% received 2.5 mg, 20.6% received 5 mg, and 12.7% received 10 mg before discontinuation. Sensitivity analysis of the dose titration patterns in the subgroup of patients that continued vericiguat for more than three months showed better up-titration and continuation on day 365 (Fig. 2, A, right panel). Multivariable logistic regression analysis revealed that hypertension (OR [95% confidence interval (CI)]: 1.54 [1.06–2.24]), dual therapy (1.89 [1.22–2.93]), triple therapy (1.57 [1.04–2.37]) and quadruple therapy (1.60 [1.06–2.42]) were significantly associated with the one-year persistence of vericiguat (Table 3).

**Table 2:** Dose titration, adherence, and persistence of vericiguat for one year following initiation in overall population and subgroups based on presence of chronic kidney disease, age, and BMI.

	**Overall** **(N = 1798)**	**With chronic kidney disease** **(N = 571)**	**Age < 65 years** **(N = 391)**	**Age 65–74 years** **(N = 474)**	**Age ≥ 75 years (N = 933)**
Up-titration of vericiguat at:					
Day 90	900 (50.1)	302 (52.9)	198 (50.6)	244 (51.5)	458 (49.1)
Day 180	944 (52.5)	309 (54.1)	208 (53.2)	251 (53.0)	485 (52.0)
Day 365	941 (52.3)	303 (53.1)	207 (52.9)	251 (53.0)	483 (51.8)
Up-titration of vericiguat to the maximal daily dose at:					
Day 90	341 (19.0)	121 (21.2)	77 (19.7)	84 (17.7)	180 (19.3)
Day 180	472 (26.3)	156 (27.3)	106 (27.1)	118 (24.9)	248 (26.6)
Day 365	540 (30.0)	175 (30.6)	121 (30.9)	140 (29.5)	279 (29.9)
MPR ≥ 80%	1721 (95.7)	541 (94.7)	372 (95.1)	452 (95.4)	897 (96.1)
PDC ≥ 80%	1228 (68.3)	385 (67.4)	268 (68.5)	332 (70.0)	628 (67.3)
Continuous treatment at Day 365	1197 (66.6)	372 (65.1)	271 (69.3)	316 (66.7)	610 (65.4)

	BMI* <18.5 kg/m^2^ **(N = 139)**	**BMI** **18.5**–**24.9 kg/m^2^** **(N = 609)**	**BMI** **25.0**–**29.9 kg/m^2^** **(N = 257)**	**BMI** **≥30.0 kg/m^2^** **(N = 87)**
Up-titration of vericiguat at:				
Day 90	68 (48.9)	261 (42.9)	122 (47.5)	49 (56.3)
Day 180	66 (47.5)	270 (44.3)	118 (45.9)	49 (56.3)
Day 365	61 (43.9)	274 (45.0)	117 (45.5)	46 (52.9)
Up-titration of vericiguat to the maximal daily dose at:				
Day 90	32 (23.0)	102 (16.7)	55 (21.4)	16 (18.4)
Day 180	39 (28.1)	134 (22.0)	69 (26.8)	19 (21.8)
Day 365	39 (28.1)	148 (24.3)	79 (30.7)	27 (31.0)
MPR ≥ 80%	132 (95.0)	574 (94.3)	242 (94.2)	84 (96.6)
PDC ≥ 80%	84 (60.4)	370 (60.8)	165 (64.2)	50 (57.5)
Continuous treatment at Day 365	78 (56.1)	355 (58.3)	167 (65.0)	47 (54.0)

Data are presented as n (%). Analyses were conducted in patients with one year of follow-up (n = 1798). *Data on BMI were missing in 39.3% of patients. BMI, body mass index; MPR, medication possession ratio; PDC, proportion of days covered.

**Table 3:** Multivariable logistic regression analysis exploring factors associated with one-year persistence of vericiguat.

**Variables**	**OR (95% CI)**	*P* value*
**Age**		
65–74 years vs. < 65 years	0.91 (0.62–1.32)	0.611
≥75 years vs. < 65 years	0.77 (0.54–1.10)	0.160
**Sex,** female vs. male	0.89 (0.66–1.18)	0.413
BMI**		
Underweight vs. normal weight	0.99 (0.67–1.45)	0.940
Pre-obesity vs. normal weight	1.23 (0.90–1.69)	0.206
Obesity vs. normal weight	0.76 (0.47–1.23)	0.257
**Recent WHF event,** yes vs. no	0.82 (0.59–1.13)	0.227
**Comorbidities**		
Hypertension, yes vs. no	1.54 (1.06–2.24)	0.024
Chronic kidney disease, yes vs. no	1.03 (0.79–1.36)	0.823
Diabetes mellitus, yes vs. no	1.24 (0.94–1.63)	0.135
Atrial fibrillation, yes vs. no	1.12 (0.87–1.44)	0.396
Coronary artery disease, yes vs. no	0.91 (0.68–1.22)	0.540
Peripheral artery disease, yes vs. no	1.23 (0.90–1.68)	0.188
Stroke, yes vs. no	0.81 (0.55–1.19)	0.285
COPD, yes vs. no	0.97 (0.60–1.57)	0.898
Anemia, yes vs. no	1.19 (0.91–1.56)	0.208
**Cardiovascular procedure,** yes vs. no	1.14 (0.77–1.71)	0.518
**Dialysis,** yes vs. no	0.51 (0.20–1.28)	0.153
**HF foundational therapy**		
Dual therapy vs. monotherapy	1.89 (1.22–2.93)	0.004
Triple therapy vs. monotherapy	1.57 (1.04–2.37)	0.032
Quadruple therapy vs. monotherapy	1.60 (1.06–2.42)	0.026

Variables were selected from patient characteristics. Highly correlated variables were removed based on the correlation matrix. Patients without missing variables (n = 1,092) were included in this analysis. *Wald test. **BMI was categorized as underweight (<18.5 kg/m^2^), normal weight (18.5–24.9 kg/m^2^), pre-obesity (25.0–29.9 kg/m^2^), and obesity (≥30.0 kg/m^2^). BMI, body mass index; CI, confidence interval; COPD, Chronic obstructive pulmonary disease; HF, heart failure; OR, odds ratio; WHF, worsening heart failure.

### 3.4 Changes in laboratory and clinical parameters before and after vericiguat initiation

**Fig. 3:**
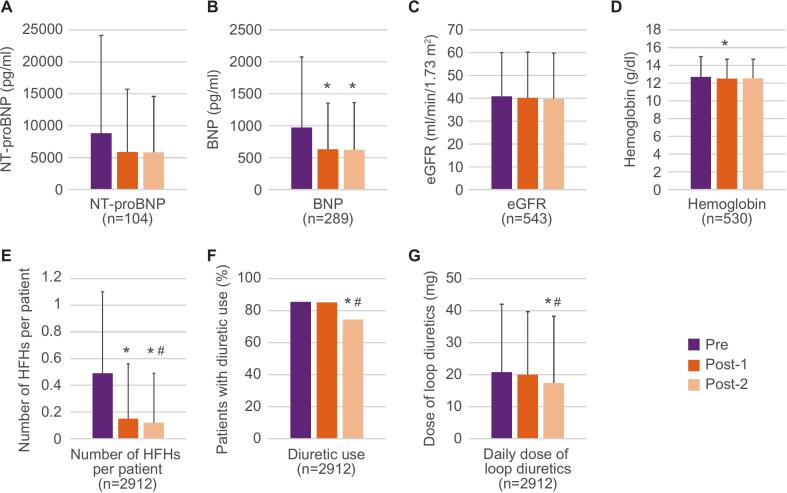
Changes in laboratory and clinical parameters before and after vericiguat initiation. A, NT-proBNP, B, BNP, C, eGFR, D, hemoglobin, E, number of HFHs per patient, F, diuretic use, and G, daily dose of loop diuretics were assessed at pre- (day −90 to day −1) and post-vericiguat initiation periods (post-1, day 1 to day 90; post-2, day 91 to day 180). Bars indicate standard deviations for each mean value. *Adjusted p < 0.05 vs pre. #Adjusted p < 0.05 vs post-1. BNP, B-type natriuretic peptide; eGFR, estimated glomerular filtration rate; HFH, heart failure hospitalization; NT-proBNP, N-terminal pro-B-type natriuretic peptide.

Among the 530 facilities included in the dataset used in this study, 110 provided laboratory test results. The mean NT-proBNP and BNP values before vericiguat initiation (pre, day −90 to day −1) were 8835 (SD 15334) pg/ml (median 4692 pg/ml; 1st quartile, 1768 pg/ml; 3rd quartile, 11,351 pg/ml) and 974 (SD 1102) pg/ml (median 599 pg/ml; 1st quartile, 288 pg/ml; 3rd quartile, 1182 pg/ml), respectively. Compared with the period before vericiguat initiation (pre), significant decreases were observed in BNP, hemoglobin, number of HFHs per patient, diuretic use, and daily dose of loop diuretics after initiation (post-1, day 1 to day 90 or post-2, day 91 to day 180) (Fig. 3). Although no significant difference was observed in NT-proBNP levels, a numerical decreasing trend was observed. Additionally, eGFR remained stable. A similar trend was observed among patients whose HF therapeutics remained unchanged, except for the daily dose of loop diuretics, which showed no significant changes (Supplementary Fig. 1).

### 3.5 Clinical outcomes

The one-year cumulative incidence of composite of CV death or HFH calculated using the Kaplan–Meier method was 29.8 (95% CI: 28.3–31.4) %. The one-year cumulative incidences of CV death, HFH, composite of all-cause death or HFH, and all-cause death were 7.0 (6.2–8.0), 28.9 (27.3–30.5), 31.9 (30.3–33.5), and 9.9 (8.9–11.0) %, respectively. CV death accounted for 70% of all all-cause deaths. The incidence rate of composite of CV death or HFH was 40.5 events/100 patient-years. The incidence rates of CV death, HFH, composite of all-cause death or HFH, and all-cause death were 7.6, 38.8, 43.7, and 10.8 events/100 patient-years, respectively. The mean follow-up period for composite events and HFH was 193 (SD 137) days, and that for CV death and all-cause death was 225 (SD 135) days (Table 4).

**Table 4:** Clinical outcomes.

	**Number of event,** **n**	**Follow-up period,** **mean days (SD)**	**Incidence rate,** **events/100 patient-years**	**One-year cumulative incidence,** **% (95% CI)**
CV death or HFH	1059	193 (SD 137)	40.5	29.8 (28.3–31.4)
CV death	231	225 (SD 135)	7.6	7.0 (6.2–8.0)
HFH	1014	193 (SD 137)	38.8	28.9 (27.3–30.5)
All-cause death or HFH	1140	193 (SD 137)	43.7	31.9 (30.3–33.5)
All-cause death	328	225 (SD 135)	10.8	9.9 (8.9–11.0)

CI, confidence interval; CV, cardiovascular; HFH, heart failure hospitalization; SD, standard deviation; WHF, worsening heart failure.

### 3.6 Subgroups of interest

**Fig. 4:**
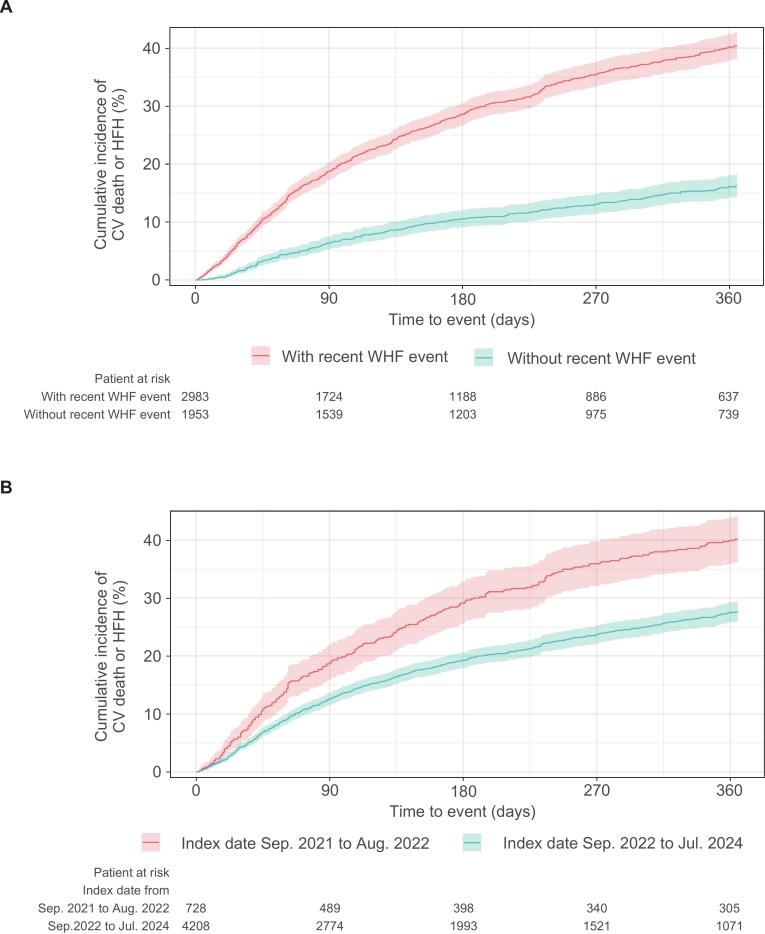
Cumulative incidence of CV death or HFH in subgroups based on A, the presence or absence of a recent WHF event, and B, index date. The shaded area represents the 95% confidence interval. CV, cardiovascular; HFH, heart failure hospitalization; WHF, worsening heart failure.

In this study, 39.6% of new vericiguat users did not have a recent WHF event at baseline; however, this population was not part of the phase III VICTORIA trial [6], [8]. Therefore, subgroup analyses based on the presence or absence of a recent WHF event were conducted to examine the usage patterns of vericiguat and the patient prognosis in each group. Compared numerically with patients with a recent WHF event, patients without a recent WHF event had a lower mean age, a higher rate of vericiguat initiation in an outpatient setting, a greater number of patients who were up-titrated, better persistence, and a lower cumulative incidence of clinical outcomes (Supplementary Tables 5–6 and Fig. 4, A). Regarding the composite of CV death or HFH, the HR adjusted for age and sex was 2.95 (95% CI: 2.55–3.40) for patients with a recent WHF event versus those without, and additional adjustments for comorbidities (hypertension, chronic kidney disease, coronary artery disease, atrial fibrillation, diabetes mellitus, stroke, anemia, chronic obstructive pulmonary [COPD], and peripheral artery disease) did not materially change this HR (Supplementary Table 7).

Considering the 14-day prescription limit during the first year after commercial launch in Japan, subgroup analyses based on index date were conducted. A recent WHF event at baseline was present in 72.4% and 58.4% of patients in the early and late groups, respectively (Supplementary Table 5). Compared numerically with the early group, the late group demonstrated better adherence, persistence, and prognosis (Supplementary Table 6 and Fig. 4, B).

Subgroups based on up-titration, adherence, and persistence of vericiguat were defined by the status during the initial 90-day period. Landmark analysis after day 90 demonstrated that the subgroups with favorable adherence or persistence tended to have a lower cumulative incidence of CV death or HFH. However, the 95% CI overlapped across the subgroups, and no clear differences were observed (Supplementary Table 8 and Supplementary Fig. 2).

Factors such as worsening renal function, older age, and low BMI are known to be associated with non-use or sub-target dosing of HF therapies [19]. In the present study, no obvious differences were observed in the dosage, adherence, and persistence of vericiguat among subgroups based on presence of chronic kidney disease, age, and BMI (Table 2).

## 4 Discussion

Using a nationwide hospital administrative database that includes more than 48 million patients cared for at 530 acute care centers as of July 2024, representing about 30% of all acute care centers in Japan, we delineated real-world prescribing patterns and cardiovascular outcomes over one year after vericiguat initiation. The strengths of this study include a large cohort size derived from an unselected, real-world population of vericiguat users.

### 4.1 Characteristics of patients initiating vericiguat

The findings of the present study demonstrated that, in real-world clinical practice, vericiguat is administered to a broader patient population than that included in the VICTORIA trial [6], [8], [20]. Compared with the VICTORIA trial, the patients in this study were older and had a higher prevalence of comorbidities, indicating a higher risk profile. This observation is consistent with findings from other real-world studies in Germany, Spain, and China, which similarly reported the use of vericiguat in a wider patient spectrum compared to the VICTORIA trial [11], [12], [13], [14]. In addition, the present study included patients both with and without a recent WHF event at baseline. Patients without a recent WHF event correspond to those evaluated in the VICTOR trial [21], [22], [23], the results of which had not been published when our study was conducted; however, they were not included in the VICTORIA trial. The inclusion of these patients in this study can be attributed to the following: First, the approved indication for vericiguat in Japan is for chronic heart failure in patients receiving standard therapy and is not restricted to patients with a recent WHF event, unlike the enrichment criteria used in the VICTORIA trial. Second, our definition of recent WHF event was based on HFH within 6 months or outpatient IV diuretics within 3 months, which may not capture other clinically relevant deterioration, for example, oral diuretic escalation, urgent visits, biomarker worsening, or events treated outside the reporting facility. As a result, some patients categorized as “no recent WHF event” may have had clinically meaningful worsening not captured by these definitions. Third, initiation in this group likely reflects clinician-identified high-risk or early worsening features in routine care, which may introduce confounding by indication that warrants cautious interpretation of between-group comparison. Recently, machine learning has been used to cluster disease phenotypes [24]. Similar methods could potentially be applied in the future to identify clinical phenotypic presentations associated with subsequent exacerbation, thereby enabling better preventive interventions.

### 4.2 Vericiguat usage patterns

Our results indicated that vericiguat was administered before the four foundational therapies in the vast majority of patients, and that multiple HF medications were introduced and escalated within relatively short period around vericiguat initiation. These findings may reflect the influence of recent literature, such as the STRONG-HF trial [25], American and European guidelines [3], [4], [26], the ESC HFA consensus document/statement [27], [28], and a review on WHF management [29], which support the early initiation of a multidrug approach with distinct mechanisms of action, as well as an individualized strategy tailored to patient characteristics. A previous study in Germany reported that the proportion of patients receiving quadruple therapy increased from 29% to 44% after vericiguat initiation [11]. Similarly, the present study showed a slight increase in the proportion of patients with a prescription record for quadruple therapy, from 32% (Day −90 to −1) to 35% (Day 0 to 90) (Fig. 1, C). On the other hand, the day-to-day analysis showed a decrease in dosage for each medication after vericiguat initiation (Fig. 1, A), which is likely because the number of patients who discontinued or down-titrated the medications exceeded the number of those who initiated or up-titrated them.

While our findings align with other real-world studies in demonstrating a broader use of vericiguat compared to the VICTORIA trial, a noteworthy distinction emerges when comparing study designs. Facility-based registry studies generally reported higher rates of baseline foundational therapies [12], [13], [14] and greater rates of vericiguat up-titration and persistence [14] compared to retrospective database studies, such as ours and the German study [11]. These differences may be attributable to a form of selection bias; centers that participate in registries may have a greater commitment to, or expertise in, optimizing heart failure therapy, potentially leading to more proactive treatment patterns.

Challenges persist in the continuation of HF medications in real-world clinical practice, and discontinuation often occurs in the early stages after initiating the therapy [30]. Our study found that 66.6% of patients continued vericiguat treatment one year after its initiation. In a previous study involving Japanese patients hospitalized for HF, the one-year continuation rates for newly initiated ACEi, ARB, beta-blocker, MRA and SGLT2i were 50.8%, 44.2%, 50.5%, 40.7%, and 59.8%, respectively [31]. Both studies indicate that despite some differences in continuation rates among various medications, significant challenges remain in the continuation of HF treatment in real-world settings. Furthermore, vericiguat discontinuation occurred frequently in the early stages after initiation. Sensitivity analysis indicated favorable up-titration and continuation rates in patients who continued vericiguat for more than three months. The presence of hypertension and the use of dual, triple, and quadruple therapy versus monotherapy were significantly associated with the one-year persistence of vericiguat. Patients with hypertension may more easily tolerate multiple HF medications with blood pressure-lowering effects, which could facilitate the continuation of their medication regimen. While the reasons for discontinuation could not be obtained from this database, the potential factors may include issues related to tolerability, changes in treating physicians owing to discharge or transfer, lack of patient understanding of the necessity for continued treatment, and clinical inertia. To help avoid vericiguat discontinuation unless tolerability issues arise, particularly early in treatment, effective communication with post-discharge healthcare providers and patient education may support continued use and dose escalation, potentially improving outcomes.

### 4.3 Outcomes

As mentioned previously, this study included a broader patient population than the VICTORIA trial. Notably, the cumulative incidence of composite of CV death or HFH was largely similar to that observed in the VICTORIA trial [8]. This could be because the higher risk associated with the older age and greater comorbidity burden in our cohort may have been offset by the inclusion of a substantial proportion of patients without a recent WHF event. On the other hand, our findings indicate that the one-year cumulative incidences of CV death and all-cause death in our study were lower than those reported in the VICTORIA trial, while the one-year cumulative incidence of HFH was higher. This finding may be attributed to our broader definition of HFH, which was based on secondary data usage, compared to the more stringent criteria used in the clinical trial. Additionally, differences in healthcare systems and hospitalization practices across countries may also influence the frequency of HFH, highlighting the need to consider these factors when interpreting the results.

It may be worth mentioning that renal function remained stable after vericiguat initiation. Additionally, natriuretic peptide levels and diuretic use were decreased. These findings are complemented by those from the VERITA study, a prospective observational study in Spain, which reported improvements in New York Heart Association functional class and EuroQol-5D, and a decrease in the number of HF-related hospitalization/decompensations after vericiguat initiation [14]. Although no firm conclusions regarding causality can be established due to the single-arm study design, the findings from both studies, assessing different sets of parameters, suggest potential benefits with vericiguat in a real-world setting.

Consistent with previous findings [2], [32], [33], our results confirm the considerable impact of a recent WHF event on patient prognosis. Furthermore, in the present study, 40% of patients had no recent WHF event, which corresponds to the population evaluated in the VICTOR trial [21], [22], [23], making it one of the first large-scale real-world studies on vericiguat in this specific group. The patterns of vericiguat use in daily clinical practice have evolved over time, with the late group demonstrating better continuation rates, adherence, and prognosis compared with the early group. The lifting of the two-week prescription limit for vericiguat in September 2022, which allowed for long-term prescriptions alongside the expansion of prescriptions to lower-risk patients and the accumulation of prescribing experience, may have contributed to these changes. Although a trend toward a lower cumulative incidence of CV death or HFH was observed in subgroups with favorable adherence or persistence, it is difficult to conclude a definitive relationship between the administration status of vericiguat and patient outcomes. This difficulty arises from the retrospective observational design of this study, which is susceptible to potential selection bias and confounding among subgroups. For instance, unrecorded clinical rationales likely influenced treatment decisions such as up-titration or discontinuation. Prospective studies, similar to the SOCRATES-REDUCED trial [34], are necessary to clarify this relationship.

### 4.4 Study limitations

This study has several limitations. First, the generalizability of the findings may be restricted when considering other countries with different healthcare systems or local guidelines for HF treatment. Second, although the MDV data included many patients across various geographical regions and age groups in Japan, the data was primarily collected for reimbursement rather than research purposes. Consequently, the entire patient journey may not be fully captured, particularly if patients were transferred to other hospitals or received care from different medical institutions. Although it is likely that most patients had HFrEF given the recommendation for vericiguat in Japan, this study could not confirm this diagnosis for the entire cohort, as ejection fraction data were available for only a subset of patients and the Japanese ICD-10 system lacks a specific code for HFrEF. Deaths in the MDV database were based on patient discharge records from hospitals; thus, data on outpatient mortality are lacking. Additionally, as the laboratory test results were obtained from a limited number of medical institutions, these findings may not be generalizable to a broader population. Lastly, the reasons behind the lack of dose up-titration or discontinuation could not be ascertained, as the information was not captured in the MDV database.

## 5 Conclusions

The results of the ROVER-Japan study demonstrate that vericiguat was initiated across a broad spectrum of HF patients, most of whom were not receiving quadruple HF therapy. Natriuretic peptide levels and diuretic use decreased, while eGFR remained stable after vericiguat initiation. The one-year cumulative incidence of composite of CV death or HFH was largely similar to that observed in the VICTORIA trial. Prescription patterns evolved to include a higher proportion of patients without a recent WHF event, suggesting growing clinical familiarity. Consistent use patterns across subgroups based on presence of chronic kidney disease, age, and BMI support the broad applicability of vericiguat for complex HF patients. These findings complement existing trial evidence and provide clinicians with contemporary data on vericiguat use in everyday practice.

## CRediT authorship contribution statement

**Makiko Takeichi:** Writing – review & editing, Writing – original draft, Conceptualization. **Shun Kohsaka:** Writing – review & editing, Conceptualization. **Kotaro Nochioka:** Writing – review & editing, Conceptualization. **Christoph Ohlmeier:** Writing – review & editing, Conceptualization. **Alexander Michel:** Writing – review & editing, Conceptualization. **Katsiaryna Holl:** Writing – review & editing, Conceptualization. **Hiroki Yamamoto:** Writing – review & editing, Formal analysis, Data curation. **Yoshifumi Arita:** Writing – review & editing, Formal analysis, Data curation. **Seok-Won Kim:** Writing – review & editing, Formal analysis, Data curation. **Suguru Okami:** Writing – review & editing, Conceptualization.

## Funding

This study was funded by Bayer AG (Leverkusen, Germany). All authors from Bayer were involved in the study design, interpretation of results, preparation of the manuscript, and decision to submit the manuscript for publication.

## Declaration of competing interest

The authors declare the following financial interests/personal relationships which may be considered as potential competing interests: Suguru Okami reports financial support was provided by Bayer AG. Suguru Okami reports a relationship with Bayer Yakuhin, Ltd. that includes: employment. Makiko Takeichi reports a relationship with Bayer Yakuhin, Ltd. that includes: employment. Shun Kohsaka reports a relationship with JSPS that includes: funding grants. Shun Kohsaka reports a relationship with Pfizer that includes: funding grants. Shun Kohsaka reports a relationship with Bayer that includes: consulting or advisory and speaking and lecture fees. Shun Kohsaka reports a relationship with Bristol Myers Squibb that includes: speaking and lecture fees. Shun Kohsaka reports a relationship with Novartis that includes: speaking and lecture fees. Kotaro Nochioka reports a relationship with Bayer Yakuhin, Ltd. that includes: speaking and lecture fees. Kotaro Nochioka reports a relationship with AstraZeneca that includes: speaking and lecture fees. Kotaro Nochioka reports a relationship with Eli Lilly Japan K.K. that includes: speaking and lecture fees. Kotaro Nochioka reports a relationship with Amicus Therapeutics that includes: speaking and lecture fees. Kotaro Nochioka reports a relationship with Sanofi that includes: speaking and lecture fees. Christoph Ohlmeier reports a relationship with Bayer AG that includes: employment. Katsiaryna Holl reports a relationship with Bayer AG that includes: employment. Alexander Michel reports a relationship with Bayer Consumer Care AG that includes: employment. Alexander Michel reports a relationship with Bayer AG that includes: equity or stocks. Yoshifumi Arita reports a relationship with AstraZeneca K.K. that includes: employment. Yoshifumi Arita reports a relationship with IQVIA Solutions Japan G.K. that includes: employment. Hiroki Yamamoto reports a relationship with IQVIA Solutions Japan G.K. that includes: employment. Seok Won Kim reports a relationship with IQVIA Solutions Japan G.K. that includes: employment. Given their role as Associate Editor, Kotaro Nochioka had no involvement in the peer review of this article and had no access to information regarding its peer review. Full responsibility for the editorial process for this article was delegated to another journal editor.
