# Supplementary material for: One-year real-world outcomes after vericiguat initiation in heart failure: The ROVER-Japan cohort study
Source: Int J Cardiol Heart Vasc. 2026 Mar 13;63:101900. doi: 10.1016/j.ijcha.2026.101900 (PMC12996802; doi:10.1016/j.ijcha.2026.101900)

Supplementary Table 1. Inclusion and exclusion criteria

| Inclusion Criteria |
| --- |
| - Patients who initiated vericiguat between September 15, 2021, and July 31, 2024 - Patients with a diagnosis code of HF between March 1, 2021, and July 31, 2024, before the index date - Patients who initiated vericiguat at a dose of 2.5 mg/day - Patients who received any dose of at least one HF foundational therapy (i.e., ACEi, ARB, ARNI, beta-blocker, MRA, and SGLT2i) within 90 days before the index date - Patients aged ≥18 years at the index date |
| Exclusion Criteria |
| - Patients with observability for less than 180 days prior to the index date - Patients with a follow-up period of 0 days |
| Index date: date of vericiguat initiation. ACEi, angiotensin-converting enzyme inhibitor; ARB, angiotensin receptor blocker; ARNI, angiotensin receptor-neprilysin inhibitor; HF, heart failure; MRA, mineralocorticoid receptor antagonist; SGLT2i, sodium-glucose cotransporter 2 inhibitor. |
|  |

Supplementary Table 2. Variable definitions

| Diseases (covariate) | ICD-10 code | Kubun code/Receipt code |
| --- | --- | --- |
| HF | I50, I11.0 |  |
| Stroke | I60-I63 |  |
| Peripheral artery disease | E10.5, E11.5, E13.5, E14.5, I65.2, I70, I73, I74, I77.1 |  |
| Chrinic kidney disease | N18 | Kubun code:  K780, K780-2, J038, J038-2  Receipt code:  140007710, 140007910, 140008170, 140033770, 140036710, 140051010, 140051110, 140052570, 140052810, 140052970, 140055970, 140057810, 140057910, 140058010, 140058110, 140058210, 140058310, 140058410, 140058510, 140058610, 140058770, 140058870, 140058970, 140059070, 140059170, 140059310, 140059410, 140059510, 140060210, 140060310, 140060410, 140060510, 140060610, 140060710, 140060810, 140060910, 140061010, 140062770, 140062870, 150196310, 150196410, 150196570, 150324810, 150338610, 150419370, 150420970, 150421070 |
| Hypertension | I10–13, I15, I67.4 |  |
| Coronary artery disease | I20–I25 |  |
| Atrial fibrillation | I48.0-I48.2, I48.9  *For I48.9, it was limited to those that include the term "atrial fibrillation". |  |
| Diabetes mellitus | E10-E14 |  |
| Myocardial infarction | I21–23, I25.2  *The definition of MI differed between the covariate and clinical outcome. |  |
| Anemia | D50-53, D55-59, D60-64 |  |
| Hyperkalemia | E87.5 |  |
| Chronic obstructive pulmonary disease | J44 |  |
| Procedures | Kubun code | Receipt code |
| Biventricular pacemaker | K598, K598-2, K599-3, K599-4 | 150303210, 150415210, 150322210, 150415410, 150415110, 150415310, 150336910, 150337010, 150415910, 150416110, 150415810, 150416010, 710010059, 710010690, 710010743, 710010744, 710010759, 710010760, 710010761, 710010762, 710010858, 710010859, 710011098, 710011099, 710011100, 710011101 |
| Implantable cardioverter defibrillator | K599, K599-2, K599-3, K599-4 | 150387410, 150336910, 150275310, 150275210, 150337010, 150415710, 150415910, 150383250, 150416110, 150415810, 150415610, 150415510, 150416010, 710010059, 710010658, 710010690, 710010737, 710010738, 710010739, 710010740, 710010741, 710010742, 710010743, 710010744, 710010759, 710010760, 710010761, 710010762, 710010858, 710010859, 710010890, 710011098, 710011099, 710011100, 710011101, 730660000, 732210000 |
| Dialysis (maintenance dialysis) |  | 113002510, 140036710, 140051010, 140051110, 140052810, 140057810, 140057910, 140058010, 140058110, 140058210, 140058310, 140058410, 140058510, 140058610, 140059170, 140059310, 140059410, 140059510, 140060210, 140060310, 140060410, 140060510, 140060610, 140060710, 140060810, 140060910, 140061010, 190167970 |
| Medications | WHO ATC code | Receipt code |
| Vericiguat | C01DX22 |  |
| ACEi | C09AA02 (enalapril)  C09AA03 (lisinopril) |  |
| ARB | C09CA06 (candesartan) |  |
| ARNI | C09DX04 (sacubitril-valsartan) |  |
| Beta-blocker | C07AB07 (bisoprolol)  C07AG02 (carvedilol)  *Bisoprolol patches were exluded. |  |
| MRA | C03DA01 (spironolactone)  C03DA04 (eplerenone) |  |
| SGLT2i | A10BK01 (dapagliflozin)  A10BK03 (empagliflozin) |  |
| Loop diuretics | C03CA01 (furosemide)  C03CA04 (torasemide) | 610433078, 610453012, 610454048, 612130353, 620270703, 622161501, 622161502, 622721300, 622721400, 622879701, 622879801 (azosemide) |
| Thiazide diuretics | C03AA03 (hydrochlorothiazide)  C03AA06 (trichlormethiazide) |  |
| Vasopressin V2 receptor antagonist | C03XA01 (tolvaptan / tolvaptan sodium phosphate) |  |
| Parameters | JLAC10 code | |
| NT-proBNP | 4Z272000000000000 | |
| BNP | 4Z271000000000000 | |
| eGFR | 8A065000000000000, 8A0650000000000XX  *If there are two types of records on the same day, priority was given to "8A0650000000000XX". | |
| Hemoglobin | 2A030000000000000 | |
| ACEi, angiotensin-converting enzyme inhibitor; ARB, angiotensin receptor blocker; ARNI, angiotensin receptor-neprilysin inhibitor; BNP, B-type natriuretic peptide; eGFR, estimated glomerular filtration rate; HF, heart failure; ICD-10, International Statistical Classification of Diseases and Related Health Problems 10th Revision; MRA, mineralocorticoid receptor antagonist; NT-proBNP, N-terminal pro-B-type natriuretic peptide; SGLT2i, sodium-glucose cotransporter 2 inhibitor. | | |

Supplementary Table 3. List of dosage categories for each HF medication

| Drug | Target Maximum Dose for HF | Maximal Dose | Intermediate Dose | Low Dose |
| --- | --- | --- | --- | --- |
| ACEi |  |  |  |  |
| Enalapril | 10 mg daily | ≥10 mg daily | 5–<10 mg daily | <5 mg daily |
| Lisinopril | 10 mg daily | ≥10 mg daily | 5–<10 mg daily | <5 mg daily |
| ARB |  |  |  |  |
| Candesartan | 8 mg daily | 8-12 mg daily | 4 mg daily | 2 mg daily |
| ARNI |  |  |  |  |
| Sacubitril/Valsartan | 97 mg sacubitril and 103 mg valsartan twice daily | 97 mg sacubitril and 103 mg valsartan twice daily | 49 mg sacubitril and 51 mg valsartan twice daily | 24 mg sacubitril and 26 mg valsartan twice daily |
| BB |  |  |  |  |
| Bisoprolol | 5 mg daily | >2.5–5 mg daily | >1.25–2.5 mg daily | ≤1.25 mg daily |
| Carvedilol | 10 mg twice daily | >10 mg daily | >5–10 mg daily | ≤5 mg daily |
| MRA |  |  |  |  |
| Spironolactone | 100 mg daily | 50–100 mg daily | 25 mg daily | 12.5 mg daily |
| Eplerenone | 50 mg daily | ≥50 mg daily | 25 mg daily | <25 mg daily |
| sGC stimulator |  |  |  |  |
| Vericiguat | 10 mg daily | ≥10 mg | 5–<10 mg | <5 mg |
| Drug | HF dose | Higher dose | HF dose | Lower dose |
| SGLT2i |  |  |  |  |
| Dapagliflozin | 10 mg/day | >10 mg/day | 10 mg/day | <10 mg/day |
| Empagliflozin | 10 mg/day | >10 mg/day | 10 mg/day | <10 mg/day |
| ACEi, angiotensin-converting enzyme inhibitor; ARB, angiotensin receptor blocker; ARNI, angiotensin receptor-neprilysin inhibitor; HF, heart failure; MRA, mineralocorticoid receptor antagonist; sGC, soluble guanylate cyclase; SGLT2i, sodium-glucose cotransporter 2 inhibitor. | | | | |

Supplementary Table 4. Clinical outcome definitions

| Clinical outcomes |  |
| --- | --- |
| CV death | CV death was defined as meeting at least one of the following criteria during the follow-up period:   - Confirmed disease code for sudden cardiac death (ICD-10: I46.1). - Unknown cause of death code (ICD-10: R96, R98, R99). - Fulfilling the following two conditions in the discharge summary information (FF1Data in MDV):   - Death derived from the discharge [outcome] with ‘6. Death’ or ‘7. Death other than 6’.   - Myocardial infarction (ICD-10: I21, I22, I23), HF (ICD-10: I50, I11.0), stroke (ICD 10: I60, I61, I62, I63), pulmonary embolism (ICD-10: I26), peripheral artery disease (ICD-10: E10.5, E11.5, E13.5, E14.5, I65.2, I70, I73, I74, I77.1), or aortic dissection (ICD-10: I71) derived from the record in one of the following: ICD-10 code of the main disease name [icd10code1], ICD-10 code of the disease name behind hospitalization [icd10code2], or ICD-10 code of the disease name which input the most medical resources [icd10code3].   Death date ([dischargingdate] if [outcome] was death from ‘FF1Data’, [fromdate] from ‘DiseaseData’) was used as the event date if any CV death was observed. |
| All-cause death | - Confirmed disease code for sudden cardiac death (ICD-10: I46.1). - Unknown cause of death code (ICD-10: R96, R98, R99). - Death derived from the discharge [outcome] with ‘6. Death’ or ‘7. Death other than 6’ using the discharge summary information of the ‘FF1Data’ in MDV during the follow-up period.   Only inpatient death was considered since no data for outpatient death was available in the data source. |
| HFH | HF-related hospitalization was identified as a patient assigned HF disease codes [icdcode] of ‘I50’ or ‘I11.0’ as the [dpcdiseasesegment] codes of #01, #11 or #21 in ‘DiseaseData’ during follow-up period.   - #01: Disease requiring the highest medical resources during the hospitalization - #11: Primary disease for the hospitalization - #21: Disease that triggered the hospitalization   The date of the first HFH ([admittingdate] from ‘FF1Data’) was used as the event date. |
| Composite of CV death or HFH | The earliest date of CV death and HFH was used as the event date |
| Composite of all-cause death or HFH | The earliest date of all-cause death and HFH was used as the event date |
| CV, cardiovascular; HF, heart failure; HFH, heart failure hospitalization; ICD-10, International Statistical Classification of Diseases and Related Health Problems 10th Revision. | |

Supplementary Table 5. Subgroup patient characteristics based on the presence or absence of a recent WHF event, and index date

| Characteristic | With recent  WHF event  (N=2983) | Without recent  WHF event  (N=1953) |  | Index date Sep. 2021 to Aug. 2022  (N=728) | | Index date Sep. 2022 to Jul.2024  (N=4208) | |
| --- | --- | --- | --- | --- | --- | --- | --- |
| Age, years | 76.8 (SD 12.2) | 73.4 (SD 12.3) |  | 74.9 (SD 12.0) | | 75.5 (SD 12.4) | |
| Female sex | 1004 (33.7) | 604 (30.9) |  | 225 (30.9) | | 1383 (32.9) | |
| BMI, kg/m^2^ | 22.9 (SD 4.7) | 23.1 (SD 4.0) |  | 22.9 (SD 4.4) | | 23.0 (SD 4.6) | |
| Missing | 302 (10.1) | 1381 (70.7) |  | 162 (22.3) | | 1521 (36.1) | |
| Recent WHF event | 2983 (100) | 0 (0) |  | 527 (72.4) | | 2456 (58.4) | |
| HFH in the previous 3 months | 2672 (89.6) | 0 (0) |  | 480 (65.9) | | 2192 (52.1) | |
| HFH in the previous 3–6 months | 191 (6.4) | 0 (0) |  | 25 (3.4) | | 166 (3.9) | |
| Outpatient IV diuretics in the previous 3 months | 120 (4.0) | 0 (0) |  | 22 (3.0) | | 98 (2.3) | |
| Prior WHF event | 2983 (100) | 835 (42.8) |  | 628 (86.3) | | 3190 (75.8) | |
| Inpatient initiation of vericiguat | 2320 (77.8) | 364 (18.6) |  | 479 (65.8) | | 2205 (52.4) | |
| Baseline comorbidities |  |  |  |  | |  | |
| Hypertension | 2539 (85.1) | 1721 (88.1) |  | 636 (87.4) | | 3624 (86.1) | |
| Chronic kidney disease | 1143 (38.3) | 588 (30.1) |  | 303 (41.6) | | 1428 (33.9) | |
| Diabetes mellitus | 1897 (63.6) | 1314 (67.3) |  | 509 (69.9) | | 2702 (64.2) | |
| Atrial fibrillation | 1728 (57.9) | 1068 (54.7) |  | 446 (61.3) | | 2350 (55.8) | |
| Coronary artery disease | 1923 (64.5) | 1341 (68.7) |  | 526 (72.3) | | 2738 (65.1) | |
| Myocardial infarction | 893 (29.9) | 660 (33.8) |  | 251 (34.5) | | 1302 (30.9) | |
| Peripheral artery disease | 759 (25.4) | 512 (26.2) |  | 207 (28.4) | | 1064 (25.3) | |
| Stroke | 403 (13.5) | 240 (12.3) |  | 101 (13.9) | | 542 (12.9) | |
| COPD | 213 (7.1) | 117 (6.0) |  | 68 (9.3) | | 262 (6.2) | |
| Anemia | 1159 (38.9) | 649 (33.2) |  | 310 (42.6) | | 1498 (35.6) | |
| Hyperkalemia | 567 (19.0) | 260 (13.3) |  | 149 (20.5) | | 678 (16.1) | |
| Dialysis | 40 (1.3) | 42 (2.2) |  | 7 (1.0) | | 75 (1.8) | |
| Cardiovascular procedures |  |  |  |  | |  | |
| Biventricular pacemaker​ | 188 (6.3) | 114 (5.8) |  | 69 (9.5) | | 233 (5.5) | |
| ICD | 212 (7.1) | 155 (7.9) |  | 77 (10.6) | | 290 (6.9) | |
| HF foundational therapies |  |  |  |  | |  | |
| ACEi or ARB | 546 (18.3) | 318 (16.3) |  | 127 (17.4) | | 737 (17.5) | |
| ARNI | 1609 (53.9) | 1007 (51.6) |  | 365 (50.1) | | 2251 (53.5) | |
| ACEi, ARB, or ARNI | 1999 (67.0) | 1283 (65.7) |  | 464 (63.7) | | 2818 (67.0) | |
| Beta-blocker | 2417 (81.0) | 1637 (83.8) |  | 591 (81.2) | | 3463 (82.3) | |
| MRA | 2060 (69.1) | 1118 (57.2) |  | 487 (66.9) | | 2691 (63.9) | |
| SGLT2i | 1972 (66.1) | 1171 (60.0) |  | 430 (59.1) | | 2713 (64.5) | |
| Number of HF foundational therapies* |  |  |  |  | |  | |
| Monotherapy | 385 (12.9) | 344 (17.6) |  | 106 (14.6) | | 623 (14.8) | |
| Dual therapy | 665 (22.3) | 488 (25.0) |  | 202 (27.7) | | 951 (22.6) | |
| Triple therapy | 999 (33.5) | 595 (30.5) |  | 218 (29.9) | | 1376 (32.7) | |
| Quadruple therapy | 934 (31.3) | 526 (26.9) |  | 202 (27.7) | | 1258 (29.9) | |
| Data are presented as n (%) or mean (SD). WHF event was defined as HFH or outpatient intravenous diuretics. *HF foundational therapies: RAS inhibitor (ACEi, ARB or ARNI), beta-blocker, MRA and SGLT2i. ACEi, angiotensin-converting enzyme inhibitor; ARB, angiotensin receptor blocker; ARNI, angiotensin receptor-neprilysin inhibitor; BMI, body mass index; COPD, chronic obstructive pulmonary disease; HF, heart failure; HFH, heart failure hospitalization; ICD, implantable cardioverter defibrillator; IV, intravenous; MRA, mineralocorticoid receptor antagonist; RAS, renin-angiotensin system; SD, standard deviation; SGLT2i, sodium-glucose cotransporter 2 inhibitor; WHF, worsening heart failure. | | | | | | | |
|  | | | | |  | |  |

Supplementary Table 6. Dose titration, adherence, and persistence of vericiguat for one year following vericiguat initiation in subgroups based on the presence or absence of a recent WHF event, and index date

|  | With recent  WHF event  (N=960) | Without recent WHF event  (N=838) |  | Index date Sep. 2021 to Aug.2022  (N=438) | Index date Sep. 2022 to Jul.2024  (N=1360) |  |
| --- | --- | --- | --- | --- | --- | --- |
| Up-titration of vericiguat at: |  |  |  |  |  |  |
| Day 90 | 444 (46.3) | 456 (54.4) |  | 229 (52.3) | 671 (49.3) |  |
| Day 180 | 447 (46.6) | 497 (59.3) |  | 225 (51.4) | 719 (52.9) |  |
| Day 365 | 444 (46.3) | 497 (59.3) |  | 227 (51.8) | 714 (52.5) |  |
| Up-titration of vericiguat to the maximal daily dose at: |  |  |  |  |  |  |
| Day 90 | 182 (19.0) | 159 (19.0) |  | 117 (26.7) | 224 (16.5) |  |
| Day 180 | 233 (24.3) | 239 (28.5) |  | 123 (28.1) | 349 (25.7) |  |
| Day 365 | 257 (26.8) | 283 (33.8) |  | 134 (30.6) | 406 (29.9) |  |
| MPR ≥ 80% | 914 (95.2) | 807 (96.3) |  | 401 (91.6) | 1320 (97.1) |  |
| PDC ≥ 80% | 595 (62.0) | 633 (75.5) |  | 258 (58.9) | 970 (71.3) |  |
| Continuous treatment at Day 365 | 574 (59.8) | 623 (74.3) |  | 265 (60.5) | 932 (68.5) |  |
| Data are presented as n (%). MPR, medication possession ratio; PDC, proportion of days covered. | | | | | | |

Supplementary Table 7. Hazard ratios of clinical outcomes

|  | Crude HR  (95% CI),  with vs. without recent WHF event | *P* value^#^ | Adjusted HR  (95% CI) *,  with vs. without recent WHF event | *P* value^#^ | Adjusted HR  (95% CI) **,  with vs. without recent WHF event | | *P* value^#^ | |
| --- | --- | --- | --- | --- | --- | --- | --- | --- |
| CV death or HFH | 3.02 (2.61–3.49) | <0.001 | 2.95 (2.55–3.40) | <0.001 | 2.91 (2.52–3.37) | | <0.001 | |
| CV death | 3.46 (2.49–4.82) | <0.001 | 3.29 (2.36–4.58) | <0.001 | 3.20 (2.30–4.47) | | <0.001 | |
| HFH | 2.96 (2.55–3.42) | <0.001 | 2.89 (2.49–3.34) | <0.001 | 2.86 (2.47–3.32) | | <0.001 | |
| All-cause death or HFH | 2.78 (2.42–3.18) | <0.001 | 2.71 (2.36–3.10) | <0.001 | 2.67 (2.33–3.06) | | <0.001 | |
| All-cause death | 2.34 (1.82–3.00) | <0.001 | 2.23 (1.74–2.87) | <0.001 | 2.15 (1.68–2.76) | | <0.001 | |
| *Adjusted for age and sex. **Adjusted for age, sex, and comorbidities (hypertension, chronic kidney disease, coronary artery disease, atrial fibrillation, diabetes mellitus, stroke, anemia, COPD, and peripheral artery disease). ^#^Wald test. COPD, chronic obstructive pulmonary disease; CI, confidence interval; CV, cardiovascular; HFH, heart failure hospitalization; HR, hazards ratio; WHF, worsening heart failure. | | | | | | | | |
|  | | | | | | | |  |
|  | | | | | |  |  |  |

Supplementary Table 8. Subgroup patient characteristics based on up-titration level of vericiguat at day 90, adherence to vericiguat within 90 days, and persistence to vericiguat within 90 days

| Characteristic | |  | Low dose  at day 90  (N=1906) | | Intermediate dose  at day 90  (N=1166) | | Maximal dose  at day 90  (N=633) | | Adherent within 90 days  (N=2800) | | Non-adherent within 90 days  (N=905) | | | Persistent within 90 days  (N=2750) | | Non-persistent within 90 days  (N=955) |
| --- | --- | --- | --- | --- | --- | --- | --- | --- | --- | --- | --- | --- | --- | --- | --- | --- |
| Age, years | |  | 74.8  (SD 12.0) | | 73.9  (SD 12.6) | | 73.5  (SD 13.0) | | 73.8  (SD 12.4) | | 75.7  (SD 12.1) | | | 73.7  (SD 12.4) | | 76.0  (SD 11.9) |
| Female sex | |  | 624 (32.7) | | 362 (31.0) | | 177 (28.0) | | 838 (29.9) | | 325 (35.9) | | | 824 (30.0) | | 339 (35.5) |
| BMI, kg/m^2^ | |  | 22.9 (SD 4.5) | | 23.5 (SD 4.9) | | 23.5 (SD 4.8) | | 23.3 (SD 4.8) | | 22.9 (SD 4.5) | | | 23.3 (SD 4.8) | | 23.0 (SD 4.5) |
| Missing | |  | 618 (32.4) | | 518 (44.4) | | 226 (35.7) | | 1178 (42.1) | | 184 (20.3) | | | 1150 (41.8) | | 212 (22.2) |
| Recent WHF event | |  | 1133 (59.4) | | 582 (49.9) | | 365 (57.7) | | 1438 (51.4) | | 642 (70.9) | | | 1415 (51.5) | | 665 (69.6) |
| HFH in the previous 3 months | |  | 1032 (54.1) | | 490 (42.0) | | 312 (49.3) | | 1232 (44.0) | | 602 (66.5) | | | 1209 (44.0) | | 625 (65.4) |
| HFH in the previous 3–6 months | |  | 66 (3.5) | | 57 (4.9) | | 32 (5.1) | | 131 (4.7) | | 24 (2.7) | | | 131 (4.8) | | 24 (2.5) |
| Outpatient IV diuretics in the previous  3 months | |  | 35 (1.8) | | 35 (3.0) | | 21 (3.3) | | 75 (2.7) | | 16 (1.8) | | | 75 (2.7) | | 16 (1.7) |
| Prior WHF event | |  | 1464 (76.8) | | 843 (72.3) | | 477 (75.4) | | 2031 (72.5) | | 753 (83.2) | | | 2001 (72.8) | | 783 (82.0) |
| Inpatient initiation of vericiguat | |  | 1039 (54.5) | | 485 (41.6) | | 274 (43.3) | | 1129 (40.3) | | 669 (73.9) | | | 1117 (40.6) | | 681 (71.3) |
| Baseline comorbidities | |  |  | |  | |  | |  | |  | | |  | |  |
| Hypertension | |  | 1666 (87.4) | | 1023 (87.7) | | 562 (88.8) | | 2497 (89.2) | | 754 (83.3) | | | 2457 (89.3) | | 794 (83.1) |
| Chronic kidney disease | |  | 654 (34.3) | | 408 (35.0) | | 241 (38.1) | | 967 (34.5) | | 336 (37.1) | | | 950 (34.5) | | 353 (37.0) |
| Diabetes mellitus | |  | 1260 (66.1) | | 778 (66.7) | | 430 (67.9) | | 1899 (67.8) | | 569 (62.9) | | | 1859 (67.6) | | 609 (63.8) |
| Atrial fibrillation | |  | 1103 (57.9) | | 653 (56.0) | | 372 (58.8) | | 1603 (57.3) | | 525 (58.0) | | | 1572 (57.2) | | 556 (58.2) |
| Coronary artery disease | |  | 1285 (67.4) | | 801 (68.7) | | 428 (67.6) | | 1905 (68.0) | | 609 (67.3) | | | 1867 (67.9) | | 647 (67.7) |
| Myocardial infarction | |  | 632 (33.2) | | 355 (30.4) | | 206 (32.5) | | 900 (32.1) | | 293 (32.4) | | | 878 (31.9) | | 315 (33.0) |
| Peripheral artery disease | |  | 520 (27.3) | | 320 (27.4) | | 150 (23.7) | | 759 (27.1) | | 231 (25.5) | | | 733 (26.7) | | 257 (26.9) |
| Stroke | |  | 253 (13.3) | | 142 (12.2) | | 86 (13.6) | | 351 (12.5) | | 130 (14.4) | | | 343 (12.5) | | 138 (14.5) |
| COPD | |  | 136 (7.1) | | 77 (6.6) | | 38 (6.0) | | 186 (6.6) | | 65 (7.2) | | | 184 (6.7) | | 67 (7.0) |
| Anemia | |  | 716 (37.6) | | 413 (35.4) | | 221 (34.9) | | 1010 (36.1) | | 340 (37.6) | | | 986 (35.9) | | 364 (38.1) |
| Hyperkalemia | |  | 317 (16.6) | | 193 (16.6) | | 116 (18.3) | | 469 (16.8) | | 157 (17.3) | | | 463 (16.8) | | 163 (17.1) |
| Dialysis | |  | 33 (1.7) | | 23 (2.0) | | 8 (1.3) | | 35 (1.3) | | 29 (3.2) | | | 38 (1.4) | | 26 (2.7) |
| Cardiovascular procedures | |  |  | |  | |  | |  | |  | | |  | |  |
| Biventricular pacemaker​ | |  | 142 (7.5) | | 74 (6.3) | | 37 (5.8) | | 190 (6.8) | | 63 (7.0) | | | 191 (6.9) | | 62 (6.5) |
| ICD | |  | 165 (8.7) | | 89 (7.6) | | 51 (8.1) | | 238 (8.5) | | 67 (7.4) | | | 233 (8.5) | | 72 (7.5) |
| HF foundational therapies | |  |  | |  | |  | |  | |  | | |  | |  |
| ACEi or ARB | |  | 355 (18.6) | | 177 (15.2) | | 108 (17.1) | | 500 (17.9) | | 140 (15.5) | | | 489 (17.8) | | 151 (15.8) |
| ARNI | |  | 999 (52.4) | | 630 (54.0) | | 377 (59.6) | | 1549 (55.3) | | 457 (50.5) | | | 1515 (55.1) | | 491 (51.4) |
| ACEi, ARB, or ARNI | |  | 1272 (66.7) | | 772 (66.2) | | 462 (73.0) | | 1938 (69.2) | | 568 (62.8) | | | 1893 (68.8) | | 613 (64.2) |
| Beta-blocker | |  | 1601 (84.0) | | 972 (83.4) | | 534 (84.4) | | 2384 (85.1) | | 723 (79.9) | | | 2345 (85.3) | | 762 (79.8) |
| MRA | |  | 1259 (66.1) | | 739 (63.4) | | 409 (64.6) | | 1808 (64.6) | | 599 (66.2) | | | 1781 (64.8) | | 626 (65.5) |
| SGLT2i | |  | 1225 (64.3) | | 730 (62.6) | | 422 (66.7) | | 1823 (65.1) | | 554 (61.2) | | | 1789 (65.1) | | 588 (61.6) |
| Number of HF foundational therapies* | |  |  | |  | |  | |  | |  | | |  | |  |
| Monotherapy | |  | 265 (13.9) | | 180 (15.4) | | 73 (11.5) | | 362 (12.9) | | 156 (17.2) | | | 357 (13.0) | | 161 (16.9) |
| Dual therapy | |  | 430 (22.6) | | 277 (23.8) | | 136 (21.5) | | 636 (22.7) | | 207 (22.9) | | | 629 (22.9) | | 214 (22.4) |
| Triple therapy | |  | 612 (32.1) | | 357 (30.6) | | 214 (33.8) | | 889 (31.8) | | 294 (32.5) | | | 863 (31.4) | | 320 (33.5) |
| Quadruple therapy | |  | 599 (31.4) | | 352 (30.2) | | 210 (33.2) | | 913 (32.6) | | 248 (27.4) | | | 901 (32.8) | | 260 (27.2) |
| Data are presented as n (%) or mean (SD). WHF event was defined as HFH or outpatient intravenous diuretics. Up-titration level was categorized based on the daily dose at 90 days after index date. Adherent to vericiguat within 90 days was defined as MPR ≥ 80%. Persistent to vericiguat within 90 days was defined as no treatment discontinuation within 90 days. *HF foundational therapies: RAS inhibitor (ACEi, ARB or ARNI), beta-blocker, MRA and SGLT2i. ACEi, angiotensin-converting enzyme inhibitor; ARB, angiotensin receptor blocker; ARNI, angiotensin receptor-neprilysin inhibitor; BMI, body mass index; COPD, chronic obstructive pulmonary disease; HF, heart failure; HFH, heart failure hospitalization; ICD, implantable cardioverter defibrillator; IV, intravenous; MPR, medication possession ratio; MRA, mineralocorticoid receptor antagonist; RAS, renin-angiotensin system; SD, standard deviation; SGLT2i, sodium-glucose cotransporter 2 inhibitor; WHF, worsening heart failure. | | | | | | | | | | | | | | | | |
|  |  | | |  | |  | |  | |  | |  |  | |  |  |


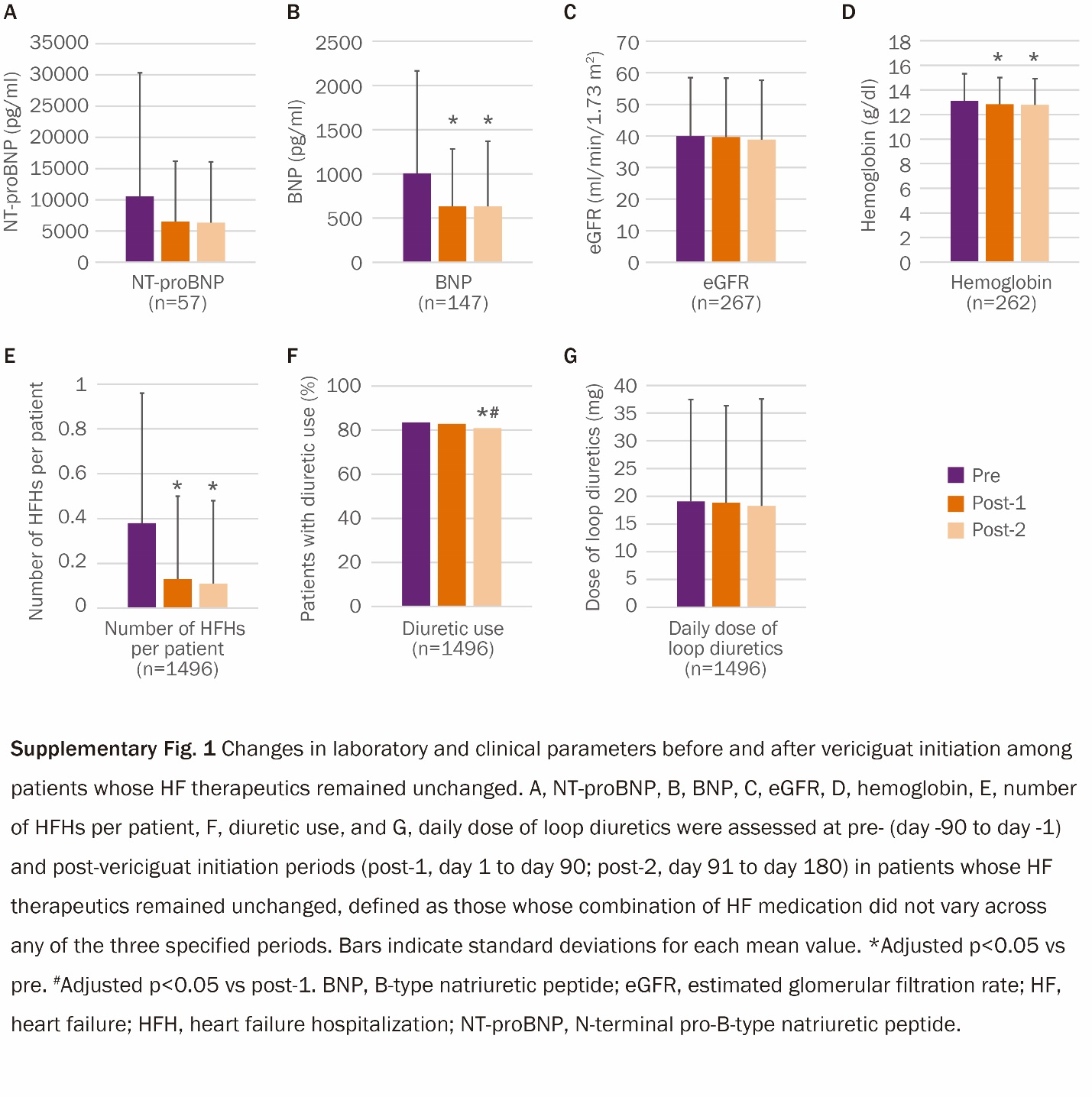


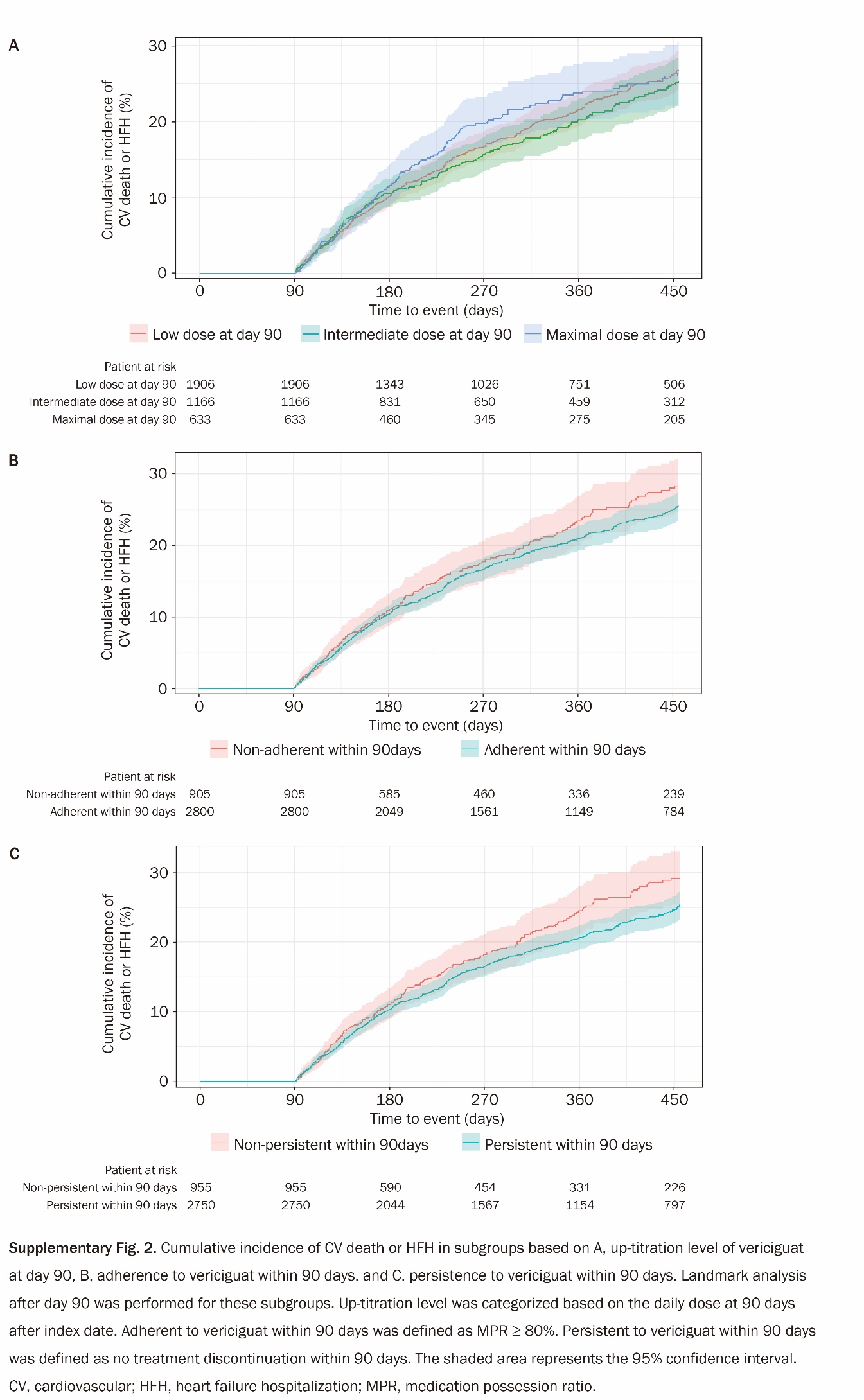

Supplement: Supplementary Data 1 [file mmc1.docx]
